# The effects of bone marrow mononuclear cell transplantation on the quality of life of children with cerebral palsy

**DOI:** 10.1186/s12955-018-0992-x

**Published:** 2018-08-14

**Authors:** Thanh Liem Nguyen, Hoang Phuong Nguyen, Trung Kien Nguyen

**Affiliations:** Vinmec Research Institute of Stem Cell and Gene Technology, 458 Minh Khai Street, Hanoi, Vietnam

**Keywords:** Quality of life, Cerebral palsy, Stem cell transplantation

## Abstract

**Background:**

Quality of life (QOL) is an important factor in evaluating the effectiveness of treatment in children with cerebral palsy (CP). The aim of this study was to evaluate the effects of autologous bone marrow mononuclear cells (BM MNCs) on the QOL of children with CP.

**Methods:**

From December 2015 to December 2016, 30 children with CP aged from 2 to 15 years received two intrathecal infusions of BM MNCs, one at baseline and the other 3 months later, at Vinmec International Hospital. The motor function and muscle tone of the patients were evaluated using the Gross Motor Function Measure (GMFM)-88 and Modified Ashworth Score, respectively. Their QOL was assessed at baseline and 6 months after the first BM MNC transplant using the Vietnamese version of the Cerebral Palsy Quality of Life Questionnaire for children (CP QOL-Child)–the parental proxy report, which comprises seven domains. Nineteen mothers (mean age: 32.9±4.9 years) and 11 fathers (mean age: 36.1±6.8 years) were invited to complete the CP QOL-Child assessment before and after the transplantations, Paired *t*-tests and multivariate regression analyses were used to evaluate the changes in QOL and GMFM scores and to identify the key factors correlated with the QOL score.

**Results:**

Significant changes were observed in the children’s gross motor function and muscle spasticity, as evidenced by the GMFM-88 total score, scores for each of its domains, the GMFM-66 percentile and the muscle tone (*P* < 0.001). Six months after the transplantations, the QOL scores of children with CP were markedly increased (*P* < 0.001) for all the domains, except for the domain of access to services. In the multivariate regression analysis, significant associations were found between higher age of children and higher QOL except for feeling about functioning and pain and impact of disability domains. Gross Motor Function Classification System (GMFCS) level was negatively correlated with the score of pain and impact of disability domain, while the GMFM-88 scores were positively correlated with the QOL in terms of feelings about functioning and family health domain (*P* < 0.05).

**Conclusion:**

The QOL of the children with CP was noticeably improved 6 months after BM MNC transplantation and was accompanied by improvements in gross motor function and muscle tone.

**Trial registration:**

ClinicalTrials.gov Identifier: NCT02574923. Registered on October 14, 2015.

## Background

Cerebral palsy (CP) is a group of permanent movement and posture development disorders [1] and one of the most common causes of motor disability in children [2–5] with an estimated overall prevalence of 2.11 per 1000 live births [6]. The motor disorders of CP are often associated with sensation difficulties, perceptual and communication *problems,* and musculoskeletal disorders [1]. As a result, the quality of life (QOL) of children with CP is severely impaired [7]. Although there is a lack of accurate prevalence data for CP in low- and middle-income countries [8], CP has been estimated to be approximately 5 to 10 times more common in underprivileged parts of the world than in developed countries [9].

Traditional CP treatments include physiotherapy, pharmacologic therapies, and botulin toxin A injection [10–13] The effectiveness of these methods, however, is limited. Recently, stem cell therapy has been found to be one of the most effective treatments for patients with neurological disorders such as spinal cord injuries, strokes, and CP in children [14–16]. Stem cell transplantation has consistently been shown to improve motor function and decrease muscle spasticity in children with CP [14, 17–19] The transplantation of autologous bone marrow mononuclear cells (BM MNCs) improved the development of children with CP [15]. However, the effects of stem cell transplantation on the QOL of children with CP and their families have rarely been reported. QOL is defined as “an individual’s perception of their position in life in the context of the culture and value systems in which they live, and in relation to their goals, expectations, standards and concerns” [20]. The evaluation of QOL has been used as an approach to achieve insight into a child’s life, to examine aspects of life that are supportive or detrimental and to provide information on the development of interventions [21].

The aim of this study was to evaluate the effects of autologous BM MNC transplantation on QOL and motor function in children with CP.

## Methods

An open label, uncontrolled clinical trial that included 2 intrathecal administrations of autologous bone marrow mononuclear cells (BM MNCs) was conducted, one at baseline and the other 3 months later. The study protocol adhered to the tenets of the the World Medical Association Declaration of Helsinki and was approved by the Vinmec International Hospital Ethics Committee. All caregivers received a written consent form, a cover letter and a clear explanation of the safety issues, potential risks and benefits, and the procedure involved.

### Participants

In total, 30 patients, including 10 girls and 20 boys with CP and their parents, were included in this study. Children with CP related to oxygen deprivation were recruited to receive BM MNCs at Vinmec International Hospital, Hanoi, Vietnam, from December 2015 to December 2016. The following inclusion criteria were used: (1) patients aged from 2 to 15 years of age who had a Gross Motor Function Classification System (GMFCS) [22] score ranging from level II to level V; (2) patients whose legal guardians provided written informed consent on behalf of the child to participate in the clinical trial; (3) patients whose legal guardians were capable of completing the questionnaires without any support; and (4) children who have no history of epilepsy, hydrocephalus with a ventricular shunt, coagulation disorders, allergies to anesthetic agents, and severe health conditions such as cancer, heart, lung, liver or kidney disorders, and active infections.

### Outcome measurements

#### Gross motor function measure (GMFM)-88

The standardized observational tool used to evaluate motor function was the Gross Motor Function Measure (GMFM)-88 [23], which consists of 88 items categorized into five domains: (1) lying and rolling; (2) sitting; (3) crawling and kneeling; (4) standing; and (5) walking, running and jumping. The GMFM-66 percentile (motor growth curve) was used to control for the effect of age on the improvement of the children’s motor function. The GMFM-66 percentile measures only the motor function of the child relative to that of other children with the same GMFCS and age. In addition, the Modified Ashworth Score [24], in which lower scores or reduced muscle tone represent motor function improvement, was applied to evaluate muscle spasticity. The primary outcomes of the motor function evaluation were the GMFM-88 and GMFM-66 percentiles, and the Modified Ashworth Score. The functional and muscle tone assessments were conducted by an experienced physiotherapist at baseline and 6 months after the first BM MNC transplant.

#### CP QOL-child questionnaire

The CP QOL-Child questionnaire was used to evaluate the QOL of the participants in our study [25]. Thirty parents were requested to complete the Vietnamese version of the CP QOL-Child questionnaire since they have a deep understanding of their children, who have difficulties expressing themselves. The QOL of children with CP was assessed at baseline and 6 months after the first BM MNC transplant using the CP QOL-Child. There are three notable features of this instrument: (1) the questionnaire was designed according to the criteria of the International Classification of Functioning, Disability and Health (ICF), (2) developed by international professionals, and (3) places an emphasis on the importance of obtaining an assessment from primary caregivers to complete the questionnaire.

This questionnaire contains 66 items referencing seven domains: (1) social well-being and acceptance; (2) feelings about functioning; (3) participation and physical health; (4) emotional well-being; (5) access to services; (6) pain and impact of disability; and (7) family health. Social well-being and acceptance refers to interactions with peers, family members and other people in the community. Social participation is a component of this domain and refers to social connectedness and relationships, the capacity for involvement in social activities, and social acceptance. Feelings about functioning refers to the ability to communicate with other people in the family and community and the ability to perform daily living activities such as feeding, dressing, and toileting. Participation and physical health refers to involvement in school activities, sports and community activities; the possession of adequate motor skills; the capacity to use aids/equipment; and bodily health and wellness. Emotional well-being refers to being happy, enjoying/being satisfied with achievements, and having a good emotional state. Access to services refers to access *to* community *services* or facilities, such as service provisions or accessibility, that support parents and children with CP. Pain and impact of disability refers to physical pain or discomfort and pain related to therapy. Family health refers to the physical and emotional well-being of parents and primary caregivers and includes parental happiness. All domains except the pain and impact of disability domain are measured on a 9-point Likert scale ranging from 1 (very unsatisfied) to 9 (very satisfied). All items in the pain and impact of disability domain are rated on a 5-point scale in which higher scores indicate a poorer condition or well-being. Therefore, before the data were analyzed, the scores for this domain were converted to the positive values [26]. The scoring procedure included two steps. First, raw scores for each item were transformed to a scale with a possible range of 0–100 points. Afterward, the domain scores were calculated by averaging the scores of all items. The Vietnamese version of the CP QOL-Child questionnaire is the first instrument to explore the QOL of children with CP in the Vietnamese population. To evaluate the appropriateness of the questionnaire for measuring the QOL of persons in the target population, the internal consistency of the Vietnamese version of the CP QOL-Child questionnaire was evaluated using Cronbach’s alpha.

### Intervention

BM MNCs were chosen for this study because stored umbilical cord blood from those patients was not available. Moreover, autologous BM MNC transplants have been reported to be effective in children with CP [15]. The intervention consisted of two intrathecal transplants of autologous BM MNCs. The first was performed at the baseline, and the second was administered 3 months later. After the first transplant, all patients underwent a 10-week rehabilitation program. Bone marrow was harvested in an operating room through the iliac crests of the patients, who were under general anesthesia. Based on our initial results in a previous study [16], the collected volume was as follows: 8 mL/kg for patients weighing less than 10 kg and [80 mL+(body weight in kg-10) × 7 mL], but not exceeding 200 mL in total, for patients weighing more than 10 kg. BM MNCs were isolated from the aspirate through the use of Ficoll density gradient centrifugation [27]. Flow cytometry was used to count and assess the viability of the BM MNCs and hematopoietic stem cells (CD34+ cells) before transplantation.

### Data analysis

Prior to any statistical analysis, the eight items in the pain and impact of disability domain that were negatively correlated with condition were converted to the positive direction so that higher scores represented a better or healthier condition [26].

A descriptive analysis was performed to characterize the study population and outcomes. Paired *t*-tests were used to evaluate the changes in total QOL score and its components before and after stem cell transplantation. Data used to examine the associations were obtained 6 months after transplantations. Multivariate regression analysis were used to examine the correlation between the domain scores and the following key factors including the GMFCS, GMFM-88 score, age of the primary caregiver, and age of children. R software version 3.4.1 was used for data analysis, and the significance level was 0.05.

## Results

### Characteristics of children with CP and their primary caregivers

In total, 30 children (10 girls and 20 boys) with CP and their parents were included in this study. The mean age of the children was 5.09±3.8 years (range: 2–15.5 years old). The majority type of CP observed in all participants was quadriplegia (29 with bilateral paresis and only one with hemiplegia); 93.4% of the children were included in level IV and level V according to the GMFCS (Table 1).

**Table 1:** Characteristics of the children with CP

Characteristic	*N* = 30 (100%)
Age (years)	
Mean±SD, range [min; max]	5.09±3.8, [2; 15.5]
≤ 5 years	20 (66.7%)
> 5 years	10 (33.3%)
Type of cerebral palsy	
Quadriplegia	29 (96.7%)
Hemiplegia	1 (3.3%)
GMFCS	
Level II	1 (3.3%)
Level III	1 (3.3%)
Level IV	13 (43.4%)
Level V	15 (50%)

Table 2 shows the characteristics of the primary guardians who completed the 66-item CP QOL-Child questionnaire. Nineteen of the 30 respondents were mothers (63%), and the other 11 were fathers. All primary caregivers were parents who were married at the time of the study, and among them, 69% of the mothers and 73% of the fathers had a vocational education or higher.

**Table 2:** Demographic characteristics of the primary caregivers

Characteristic	Mothers (*N* = 19)	Fathers (*N* = 11)
Age (years)		
Mean ± SD, range [min; max]	32.9 ± 4.9, [23; 42]	36.1 ± 6.8, [25; 55]
Marital status		
Married	100%	100%
Education level		
High school	6 (31%)	3 (27%)
Vocational training	2 (11%)	2 (19%)
Graduate	10 (53%)	5 (45%)
Postgraduate	1 (5%)	1 (9%)
Occupation		
Professional/Officer	10 (53%)	5 (45%)
Worker	2 (11%)	1 (9%)
Trader	1 (5%)	1 (9%)
Farmer	1 (5%)	1 (9%)
Others (Casual labor, Housekeeping, Cashier…)	5 (26%)	3 (28%)

### Gross motor function and muscle tone before and after stem cell transplantation

There were significant improvements in gross motor function after stem cell transplantation. The total GMFM-88 score and the scores of all component domains increased after the intervention (paired *t*-tests, *P*-value< 0.001 in all domains). There was a significant improvement in the GMFM-66 percentile (from 30 to 75%) 6 months after the BM MNC transplantation. Six months after the infusions, the muscle spasticity of the children was significantly reduced from 3.8 at baseline to 2.1 (paired *t*-test, *P*-value< 0.001). Details on the changes in gross motor function and muscle tone are shown in Table 3. There were no serious adverse events or *severe* complications during or after the transplantation procedure.

**Table 3:** Gross Motor Function Measure (GMFM) and muscle tone before and 6 months after stem cell transplantation

	Baseline	6 months posttransplantation	Mean difference
	Mean±SD	Mean±SD	[95% CI]
Total GMFM-88	21.71±10.06	40.03±14.79	18.32 [14.99; 21.64] ***
A. Lying & Rolling	16.27±9.26	37.59±17.09	21.31 [16.83; 25.79] ***
B. Sitting	12.56±10. 46	30.51±19.60	17.94 [12.20; 23.69] ***
C. Crawling & Kneeling	8.15± 6.64	22.23±19.69	14.08 [8.02; 20.13] ***
D. Standing	3.74±2.35	13.78±9.69	10.04 [4.08; 15.99] **
E. Walking, Running & Jumping	2.28±1.54	5.70±4.62	3.41 [0.22; 6.60] *
GMFM-66 Percentiles	29.76±16.68	74.96± 24.40	45.2 [35.93; 54.46] ***
Modified Ashworth Score	3.77±0.68	2.16±0.70	−1.60 [− 1.84; − 1.37] ***

***P-value < 0.0001 (paired t-test, compared to baseline)

**P-value < 0.001 (paired t-test, compared to baseline)

*P-value< 0.05 (paired t-test, compared to baseline)

### Improvement in QOL

In terms of internal consistency, Cronbach’s alpha for the parent proxy Vietnamese version of the CP QOL-Child was 0.8 (95% CI [0.7; 0.91]). The Cronbach’s alpha values, calculated by sequentially removing individual domains from the questionnaire, are presented in Table 4. As seen in the table, Cronbach’s alpha exceeded 0.8 when the domains pain and impact of disability and access to services were removed. The total correlations for the seven domains are also presented in Table 4. The correlations were acceptable (r≥0.3) for all the domains, except for the access to services domain (*r* = 0.23). Overall, the Vietnamese version of the CP QOL–Child showed good internal consistency.

**Table 4:** Internal consistency of the Vietnamese version of the CP QOL-Child (parent proxy report)

Domain of CP QOL-Child	Cronbach’s alpha^a^	Item–total correlation
Social Well-being and Acceptance	0.76	0.81
Feelings about Functioning	0.75	0.74
Participation and Physical Health	0.74	0.77
Emotional Well-being	0.76	0.81
Pain and Impact of Disability	0.82	0.44
Access to Services	0.82	0.23
Family Health	0.77	0.61
Overall		0.8 [0.7; 0.91]

^a^Cronbach’s alpha if the item is removed

The QOL scores of children with CP at baseline and 6 months after stem cell transplantation as reported by their parents are presented in Table 5. The QOL scores of children with CP after two transplants significantly increased for all the domains (*P* < 0.001), except “access to services”. Furthermore, the average scores on social well-being and acceptance, feelings about functioning, participation and physical health, emotional well-being, pain and impact of disability, and family health after two stem cell transplants were remarkably higher than the baseline values (Table 5). The “pain and impact of disability” domain showed the greatest change, with an increase of 25.6%, from 38.3 to 64%, (95% CI [19.9; 31.3]). The access to services domain decreased by an average of 3.1% from 51.9 to 48.9%, (95% CI [− 6.4; 0.2]) 6 months after the BM MNC transplantation. However, this decrease was not statistically significant (*P* = 0.067).

**Table 5:** CP QOL-Child domain scores at baseline and 6 months after stem cell transplantation

Domain of CP QOL-Child	Baseline	6 months posttransplantation	Change in QOL score	*P* value
	Mean ± SD (scores converted to 0–100)	Mean ± SD (scores converted to 0–100)	Mean [95% CI] (scores converted to 0–100)	
Social Well-being and Acceptance	49.5±12.99	61.0±10.84	11.5 [7.9; 15.1]	< 0.001
Feelings about Functioning	36.8±10.85	56.6±11.47	18.7 [14.0; 23.5]	< 0.001
Participation and Physical Health	29.5±11.62	43.0±11.41	13.5 [10.4; 16.6]	< 0.001
Emotional Well-being	46.3±18.35	69.7±12.10	23.5 [17.6; 29.3]	< 0.001
Pain and Impact of Disability	38.3±10.39	64.0±13.00	25.6 [19.9; 31.3]	< 0.001
Access to Services	51.9±9.38	48.9±8.53	−3.1 [−6.4; 0.2]	0.067
Family Health	65.4±16.14	73.1±12.24	7.7 [4.0;11.4]	< 0.001

Multivariate analysis was performed for each domain of the CP QOL-Child and included the following relevant factors: age of the primary caregivers, age of the child, GMFCS level, and GMFM – 88 total score recorded 6 months after transplantation. The results of the multivariate regression analysises are presented in Table 6. The primary caregivers’ age was not significantly correlated with QOL of children with CP, while the age of the child was positively correlated with five QOL domains. Specifically, each 2 year increase in age contributed to a 3% increase in score of three domains including social well – being and acceptance, emotional well – being, and family health, a 5% increase in score of two domains including participation and physical health and access to services. The severity of the disability, indicated by the GMFCS level, was negatively correlated with the score for the pain and impact of disability domain. For example, each additional level of GMFCS contributes to a 13% decrease in score of pain and impact of disability domain. In addition, children who had higher GMFM-88 scores were more likely to have better feelings about functioning and family health scores. Specifically, each 5 point increase in GMFM-88 contributed to 2% and 3% increase in score of family health and feelings about functioning domains, respectively.

**Table 6:** Multivariate Analysis for Domains of the CP QOL–Child and Related Factors

Variable^a^	Unit or baseline group	% Difference						
		Social Well-being and Acceptance	Feelings about Functioning	Participation and Physical Health	Emotional Well-being	Pain and Impact of Disability	Access to Services	Family Health
Age of the primary caregivers	5 years	−1.16	1.74	−4.09	−0.29	−3.71	0.78	−0.41
Age of the child	2 years	3.64*	0.36	4.63*	2.87*	1.41	5.21**	2.85*
GMFCS level	1 level	2.09	−0.07	−3.72	3.72	−13.38*	− 7.26	8.41
GMFM–88 score	5 points	0.32	3.02**	1.44	0.53	1.44	−1.16	1.75*

^*^P-value< 0.05

^**^P-value < 0.001

## Discussion

This study provides conclusive evidence of remarkable improvements in gross motor function and spasticity among children with CP 6 months after BM MNC transplantation. The findings showed that the total GMFM-88 score, all of its domains and the GMFM-66 percentile were markedly higher than those at the baseline. Our data indicate that autologous BM MNC transplantation appears to be effective in terms of increasing motor function for children with CP. Similar positive outcomes were observed in a study by Sharma and colleagues [15] that examined 71 children with incurable neurological disorders, who showed an 85% improvement in the GMFM score. Likewise, Chen et al. studied 60 patients with chronic CP who were allocated into two groups (30 patients in the transplantation group and 30 children in the control group) [18]. Motor function improved significantly in the treatment group; their GMFM scores increased 58.6% 6 months posttreatment. In addition, the reduction in muscle tone among children with CP in our study suggests that stem cell transplantation could gradually improve motor deficits and contribute to the recovery of motor function.

This is the first study to characterize the QOL of children with CP from their parents’ perspectives in a Vietnamese population. In our study, the QOL scores of individuals with CP on six domains were higher after stem cell transplantation than at baseline, particularly for the domains related to emotional well - being and pain and impact of disability. Our results are consistent with the study by Sharma et al. (2012), who reported that the administration of autologous stem cells for children with incurable neurological disorders was safe and improved their QOL [15]. Based on our results, the GMFM score was positively associated with better feelings about functioning, consistent with other studies [3, 26, 28] in which the GMFM score displayed the strongest correlation with the well-being of children with CP.

The objective of this study was to demonstrate that autologous BM MNC transplantation can change the gross motor function and QOL of children with CP. In addition to the main objective, we conducted further analyses to identify the influencing factors of QOL because they may be useful for designing tailor-made intervention programs for children with CP in the future. Association of the domain scores of CP QOL – Child with other relevant factors, including the parent’s age, child’s age, severity of the disability, and GMFM – 88 total scores after intervention were analyzed. We did not observe significant correlations between the age of the primary caregivers and all the domains of the Vietnamese CP QOL– Child. This finding was confirmed in the study by of Davis et al. (2010) and Wang et al. (2010) [26, 29]. In terms of the pain and impact of disability domain, a significant correlations were found between a lower GMFCS level and the severity of pain, as pain was the strongest predictor of a lower QOL in children and adolescents with CP [30]. Access to services was the only domain that did not improve after the intervention in this study. However, this decrease was not statistically significant. In terms of the internal consistency of the Vietnamese CP QOL– Child, as Cronbach’s alpha for the “access to services” domain was the lowest value (0.23). If this domain was removed from the questionnaire, the Cronbach’s alpha increased to 0.82, suggesting some redundancy in the scale. According to Braccialli et al. (2016), this domain is the most difficult of the questionnaire to understand [31]. Therefore, investigations employing a larger sample size and an in-depth analysis are needed in the future.. Based on our results, the determinants of QOL in children with CP were bio-psychosocial and multidimensional; the primary related factors included the age of the child, GMFCS level, and GMFM score, which have been discussed in other studies [30, 32, 33].

## Conclusions

Our study shows that BM MNC transplantation is an effective therapy for improving motor function, muscle tone and QOL in children with CP.

## Abbreviations

BM MNCs: Bone marrow mononuclear cells
CP QOL-Child: Cerebral palsy quality of life questionnaire for children
CP: Cerebral palsy
GMFCS: Gross motor function classification system
GMFM: Gross motor function measure
QOL: Quality of life
